# Large language model-driven natural language interaction control framework for single-operator bimanual teleoperation

**DOI:** 10.3389/frobt.2025.1621033

**Published:** 2025-07-17

**Authors:** Haolin Fei, Tao Xue, Yiyang He, Sheng Lin, Guanglong Du, Yao Guo, Ziwei Wang

**Affiliations:** ^1^ School of Engineering, Lancaster University, Lancaster, United Kingdom; ^2^ Department of Automation, Tsinghua University, Beijing, China; ^3^ School of Mechanical Engineering, Dalian Jiaotong University, Dalian, China; ^4^ School of Computer Science and Engineering, South China University of Technology, Guangzhou, China; ^5^ School of Biomedical Engineering, Shanghai Jiao Tong University, Shanghai, China

**Keywords:** human-robot collaboration, teleoperation, bimanual manipulation, embodied AI, large language model (LLM)

## Abstract

Bimanual teleoperation imposes cognitive and coordination demands on a single human operator tasked with simultaneously controlling two robotic arms. Although assigning each arm to a separate operator can distribute workload, it often leads to ambiguities in decision authority and degrades overall efficiency. To overcome these challenges, we propose a novel bimanual teleoperation large language model assistant (BTLA) framework, an intelligent co-pilot that augments a single operator’s motor control capabilities. In particular, BTLA enables operators to directly control one robotic arm through conventional teleoperation while directing a second assistive arm via simple voice commands, and therefore commanding two robotic arms simultaneously. By integrating the GPT-3.5-turbo model, BTLA interprets contextual voice instructions and autonomously selects among six predefined manipulation skills, including real-time mirroring, trajectory following, and autonomous object grasping. Experimental evaluations in bimanual object manipulation tasks demonstrate that BTLA increased task coverage by 76.1 % and success rate by 240.8 % relative to solo teleoperation, and outperformed dyadic control with a 19.4 % gain in coverage and a 69.9 % gain in success. Furthermore, NASA Task Load Index (NASA-TLX) assessments revealed a 38–52 % reduction in operator mental workload, and 85 % of participants rated the voice-based interaction as “natural” and “highly effective.”

## 1 Introduction

Teleoperation has emerged as a pivotal technology for controlling robotic systems in hazardous or inaccessible environments while prioritizing human safety (Moniruzzaman et al., 2022; Huang et al., 2022). It has been widely applied in space rendezvous and docking (Zhang et al., 2017; Wang et al., 2021), underwater exploration (Sun et al., 2023), and remote surgery (Bacha et al., 2022; Boehm et al., 2021). To meet the demands of these scenarios, dual-arm robotic teleoperation has gained prominence as a robust solution for executing complex tasks that require enhanced dexterity (Boehm et al., 2021; Bai et al., 2021). Unlike single-arm systems, dual-arm configurations offer superior maneuverability, increased stability, and the ability to perform asymmetric operations (Huang et al., 2022; Wu et al., 2019).

Single-person bimanual (a single operator controlling dual robotic arms) and dyad teleoperation (two operators collaboratively controlling one arm each) represent the predominant paradigms for dual-arm robotic systems. In terms of single-person teleoperation, human control performance is sensitive to hardware design ergonomics, cognitive load, and task complexity (Guo et al., 2022). The operator needs to simultaneously manage the motion and coordination of two robotic arms, which can lead to increased mental workload and reduced performance (Bai et al., 2022). Regarding dyad teleoperation, human-human communication, synchronization, and control mechanism design remain challenging in ensuring intuitive collaboration and avoiding arbitration conflict among humans (Gowrishankar et al., 2014; Huang Z. et al., 2021; Li et al., 2022). Thus, dual-arm teleoperation performance can benefit from sensory feedback, motor control, and decision-making assistance as needed. For instance, with the shared mechanism, operators can focus on performing partial tasks while the assistive agent manages the remaining (Hu Z. J. et al., 2023; Wang et al., 2024). However, existing assistance systems tend to be task-dependent or rigidly structured with fixed autonomy levels. This limits their adaptability across different scenarios and operator preferences (Clark et al., 2019; Huang Y. et al., 2021; Sena et al., 2021). These systems may struggle to handle dynamic environments or adapt to new tasks without significantly modifying the control system. Additionally, the interface between the operator and the assistive system often requires specialized training or relies on pre-programmed commands that may not be natural to users.

To address these challenges, we incorporate a large language model (LLM) into a bimanual teleoperation framework (i.e., BTLA), which combines natural language interaction with variable autonomy to support single-operator dual-arm teleoperation. BTLA enables the command of the second assistive arm through natural voice instructions. The system utilizes LLMs to interpret operator intent and select the most suitable assistance mode from a set of core manipulation skills. The main contributions of our work are summarized as follows:

1. A flexible assistance system that enables natural language control of a secondary robotic arm during bimanual teleoperation, reducing operator cognitive load while maintaining task effectiveness.
2. Integration of LLMs for robust natural language understanding in robotic control, allowing operators to command complex manipulation skills through intuitive voice instructions.
3. Comprehensive experimental evaluation demonstrating significant improvements in task performance and reduction in operator workload compared to single-operator and dyadic teleoperation.

## 2 Related works

Dual-arm teleoperation architecture can be generally categorized into two main categories: single-person bimanual (SPB) teleoperation and dual-human, dual-arm (dyadic) teleoperation. The SPB teleoperation often leads to a high mental workload for the operator, as they must manage the coordination and motion of two robotic arms in real time (Shao et al., 2020). For dyadic teleoperation, two operators collaboratively control the robotic arms, theoretically leveraging their combined expertise and cognitive capabilities (Noohi et al., 2016). developed a fundamental model for computing interaction forces during dyadic cooperative manipulation tasks. Interestingly (Che et al., 2016), found that dyadic collaboration doesn’t necessarily improve performance over individual control in teleoperation environments, highlighting the complexities of human-human coordination in robotic control (Kropivšek Leskovar et al., 2021). further investigated these dynamics by examining leader-follower relationships in human dyads during collaborative tasks, providing valuable insights into role allocation strategies. To address the challenges of coordination between operators (Li et al., 2023a), proposed a flexible system capable of dynamically switching between different control architectures and controllers during operation. Two additional routes have been widely adopted to overcome the above obstacle: (i) developing more intuitive control interfaces, and (ii) designing control assistance algorithms. Intuitive human-machine interfaces aim to provide operators with natural sensations and user-friendly means of controlling multiple-arm robots (Cheng et al., 2023). Various interface technologies have been proposed, such as gesture-based interfaces (Boehm et al., 2021), virtual reality-based interfaces (García et al., 2022), and haptic devices (Rakita et al., 2019; Li et al., 2023b), reducing the cognitive burden associated with traditional control methods. Additionally, haptic feedback algorithms (Soyguder and Abut, 2016; Cavusoglu et al., 2002; Zhou et al., 2021) have been proposed to provide force feedback to the operator, enhancing their situational awareness and control precision. Control assistance algorithms, on the other hand, focus on developing intelligent strategies to assist the operator in managing the dual-arm system, including mapping strategies that translate human input into efficient and coordinated robot motions. Shared control approaches (Zheng H. et al., 2024; Huang et al., 2022; Laghi et al., 2018; Sun et al., 2020; Huang Z. et al., 2021; Shi et al., 2024) have been introduced to combine human input with autonomous robot behaviors, assisting the operator in dual-arm manipulation tasks. Recent taxonomies have provided valuable frameworks for understanding shared control in teleoperation (Li et al., 2023c). classified shared control strategies into semi-autonomous control (SAC), state-guidance shared control, and state-fusion shared control (SFSC) based on human-autonomy interaction patterns. While developed for single-arm systems, these concepts parallel our approach—our system implements SAC-like behavior during autonomous operations and SFSC-like behavior during mirroring tasks, but extends these principles to address the unique coordination challenges of bimanual manipulation.

LLM-based methods have shown promising results in enhancing interactive capabilities of robotic systems (Zha et al., 2023; Cui et al., 2024; Singh et al., 2023). These methods leverage the strong understanding of the real world inherent in LLMs/VLMs to perform high-level planning using image cues. The planned tasks are then executed by calling upon lower-level knowledge bases for automation (Hu Y. et al., 2023; Zha et al., 2023; Li et al., 2024; Zheng Y. et al., 2024; Lin et al., 2024), allowing for more flexibility and adaptation to handle various tasks and environments. However, these LLM-based methods may not be ideal for multi-contact teleoperation and physical interaction. Object grasping and manipulation in complicated or dynamic environments may be more suitable for human operators due to their intuitive understanding of the task and the ability to adapt quickly to minor variations (Akinola et al., 2021; Balasubramanian et al., 2010). In such situations, the overhead of using an LLM for planning and automation may not justify the potential benefits. Instead of tasking the LLM with context understanding and decision-making, our approach leverages the human operator’s expertise in these areas. We utilize the LLM as a human-robot interface, concentrating on its core strength of natural language processing to effectively convey human intentions.

## 3 Methodology

We first provide the formulation of the bimanual teleoperation problem in Section 3.1. Subsequently, we present in Section 3.2 how BTLA utilizes LLM to assist humans in bimanual teleoperation tasks.

### 3.1 Problem formulation

BTLA addresses SPB teleoperation by enabling natural language control of an assistive robot arm while the operator directly manipulates the master arm. This approach allows operators to maintain precise control over critical manipulation tasks while delegating complementary actions to the assistant arm through intuitive voice commands. The assistant robot receives natural language voice instructions L (e.g., help me push the green blob together) that specify the desired assistive behavior. These instructions can be long-horizon, context-aware, or ambiguously described (e.g., move a little bit upwards), requiring sophisticated contextual understanding. At any given time t , BTLA processes multiple input streams to determine the resulting assistance behaviors. These inputs include natural language commands l that specify desired assistive behaviors, proprioceptive information from both the master arm $\left(s_{m,t}\right)$ and assistant arm $\left(s_{s,t}\right)$ , environmental observations $\left(o_{\mathrm{env,t}}\right)$ , direct human control inputs $\left(u_{t}\right)$ , and environmental sensing data $\left(z_{t}\right)$ .

Therefore, the problem formulation can be summarized as follows: given a natural language instruction l , the assistant robot’s proprioceptive information $s_{a,t}$ , the master robot’s proprioceptive information $s_{m,t}$ , human input $u_{t}$ , environment sensing information $z_{t}$ at time t and environmental observations $o_{\mathrm{env,t}}$ , the embodied AI system should generate a sequence of low-level skills from the skill base S and map them to a control policy π that enables the assistant robot to assist the human operator in performing the desired task effectively.

### 3.2 BTLA system implementation

To this end, the assistant robot must decompose the high-level instruction l into a sequence of low-level skills selected from a predefined skill base S . The chosen skills and their corresponding parameters are then mapped to a control policy π , represented by a skill function BTLA(⋅) . The skill knowledge in the skill base S can be adapted to accommodate different task requirements. Therefore, the focus of our work is not on the acquisition of these skills but rather on the effective utilization of the available skills to assist the human operator.

**FIGURE 1:**
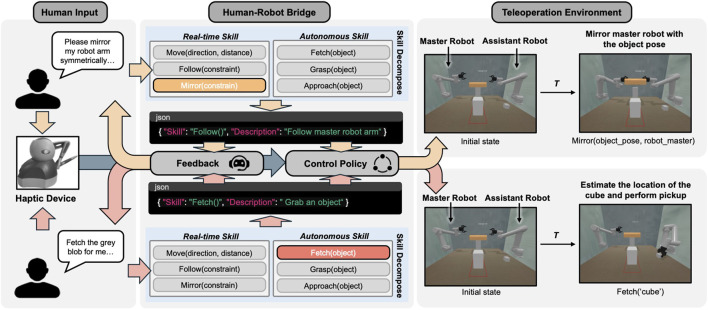
Schematic diagram of the proposed BTLA method.

BTLA consists of three key components that collaborate to enable effective assistance: (1) the natural language interface uses OpenAI’s Whisper model for speech-to-text conversion and LLM processing to interpret operator intentions; (2) a skill execution module manages the implementation of six core manipulation skills: Follow(), SymmetricalFollow(), Approach(), Move(), Handover(), and Fetch(); and (3) the control policy generator translates selected skills into robot control commands while maintaining safety constraints. Unlike a simple skill switcher, the LLM can interpret complex instructions, understand context, and provide feedback when needed. This flexibility enables the robot assistant to adapt to a wider range of scenarios and user needs, embodying the variable autonomy principle of BTLA. As shown in Figure 1, BTLA can be divided into three main components: the human operator, the human-robot interface, and the teleoperation environment. The human operator can concentrate on the current task by observing the environment via visual feedback, manipulating one robot arm with teleoperation devices, and soliciting support from the AI-assisted robot arm for collaborative task execution. The AI-assisted robot arm receives human language commands as input and identifies the most relevant skill from its skill database S , along with the necessary task parameters. The selected skill, combined with environmental data from sensors (such as visual information), proprioceptive data, and human input, forms the control policy that guides the actions of the AI-assisted robot arm. Within this configuration, the human operator collaborates with the AI-assisted robot arm within the teleoperation environment to achieve the desired task with optimal efficiency and effectiveness. The human operator provides high-level guidance and control, while the AI-assisted robot arm contributes its capabilities and understanding of the context to support the human operator in achieving their objectives. Algorithm 1 outlines the core control loop of BTLA, showing how voice commands are processed through the LLM to select and execute appropriate skills. The algorithm handles both real-time skills that require continuous execution until stopped (like following behaviors) and autonomous skills that complete specific tasks (like object fetching).

Each skill in the system is designed with clear activation conditions and completion criteria. Real-time skills like Follow() and SymmetricalFollow() maintain continuous adaptation to the master arm’s movements, while autonomous skills like Fetch() and Handover() execute specific object manipulation sequences. The system monitors execution status and provides verbal feedback to the operator, ensuring transparent operation and easy error recovery. The processing of human intent occurs in real-time while the system is executing actions. When a voice command is received, the system temporarily maintains its current action while processing the new instruction through the LLM pipeline to ensure smooth transitions between different assistance modes. The operator can issue new commands at any time, and the system will complete its current atomic action before transitioning to the new requested behavior. For safety reasons, certain commands (like “stop”) are processed with the highest priority without passing through the LLM pipeline and interrupt any ongoing action immediately.

Algorithm 1 Embodied AI-Assisted Robot Arm Control.

**Require:**Initial skills base S with predefined skills, LLM initial language description l

1: Initialize t←0 , skill←None

2: **while** not finished **do**
3:  **if** voice_command received **then**
4: skill← **LLM** (voice_command)
5: π← **BTLA** $\left(\text{skill},skill\text{\_}parameters,u_{t},z_{t}\right)$

6:   **if** skill is real-time **then**
7:    **repeat**
8:     Execute π

9: t←t+1

10:    **until** voice_command to stop
11:   **else if** skill is autonomous **then**
12:    **repeat**
13:     Execute π

14: t←t+1

15:    **until** skill is done
16:    **end if**
17:   **end if**
18: **end while**

Building upon the existing skill base and task categorization framework, our proposed system explicitly addresses scenarios involving command misinterpretations or kinematic singularities through an integrated error-handling mechanism. To ensure operational safety and task efficacy, BLTA employs a multi-stage confirmation protocol before task execution. Upon receiving an instruction, the robotic agent initiates a semantic parsing phase to interpret the command, followed by the generation of a hierarchical execution plan. This plan is then presented to the human operator via an interface for explicit validation during the execution plan verification phase, enabling cross-verification of the robot’s comprehension and providing a structured opportunity for the operator to implement necessary adjustments before deployment. Furthermore, BLTA incorporates real-time singularity detection algorithms and exception handling protocols. When kinematic singularities, operational anomalies, or unmodeled environmental constraints are detected during execution, the system initiates a suspension of operations and requests human intervention through prioritized status alerts.

Remark: This bidirectional communication framework establishes a closed-loop interaction protocol between the human operator and robotic system, enhancing system resilience through error recovery mechanisms and adaptive replanning capabilities. By integrating proactive validation checkpoints with reactive exception management, the architecture maintains optimal equilibrium between automated functionality and human supervisory control, thereby ensuring robust performance in dynamic, unstructured environments.

## 4 Experiment

**FIGURE 2:**
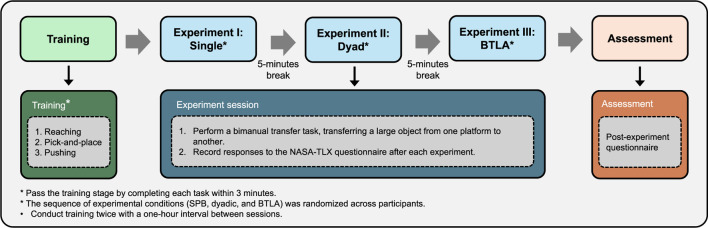
Experimental procedure for evaluating bimanual teleoperation methods. The process begins with participant training in three tasks (reaching, pick-and-place, and pushing), followed by three experimental sessions (SPB, Dyadic, and BTLA teleoperation) conducted in randomized order for each participant.

To evaluate the effectiveness of the BTLA system, we conducted experiments to move and manipulate large, heavy objects using a bimanual robotic system. The experimental procedure, from operator training to performance assessment, is illustrated in Figure 2. The assessment metrics include task efficiency, operator workload, and user satisfaction in comparison to SPB and Dyadic teleoperation methods. Ten participants (7 male, 3 female, aged 22–35) volunteered for this study, approved by Lancaster University’s Ethics Committee (FST-2024-4525-RECR-4), with informed consent obtained beforehand. All underwent comprehensive system training before testing. Participants comprised graduate students and research staff recruited from engineering and computer science disciplines. Screening confirmed that all possessed fundamental robotics literacy (e.g., coursework in control systems or human-computer interaction) but had no prior experience with bimanual teleoperation systems.

### 4.1 Experimental setup

#### 4.1.1 Equipment and software

The experimental setup incorporated two 3D Systems Touch haptic interfaces (formerly Phantom Omni). The PyBullet physics engine API was employed to construct the virtual environment, orchestrate robotic arm actuation, and render object dynamics in real time. To enhance user interface intuitiveness and operational precision, we developed a haptic feedback-enabled control architecture Equation 1 incorporating a closed-loop velocity control scheme:

$$V_{i,robot}=k_{v}d_{i,hand},$$ (1)

where i∈{x,y,z} , $V_{i,robot}$ is the velocity of the end effector of the robot arm, $d_{i,hand}$ is the displacement of the tip on the pen of the haptic device, and $k_{v}$ is the hand controller-to-robot velocity gain. The feedback force is given by Equation 2.

$$F_{i,Feedback}=k_{f}d_{i,hand}+F_{\mathrm{initial}},$$ (2)

where $F_{\mathrm{initial}}$ is the initial force that allows the user to feel a sense of boundaries. $F_{i,Feedback}$ is the feedback force on the user, which is equal in magnitude but opposite in direction to the force applied by the human on the haptic device, i.e., $F_{\mathrm{Feedback}}=-F_{\mathrm{Human}}$ . This feedback force creates a sense of resistance when the user tries to move further, allowing the user to experience greater resistance when expecting a larger robot arm moving speed. To minimize uncontrolled variables that might influence the experiment results, we designed customized objects using Fusion 360 and converted them into URDF files.

#### 4.1.2 LLM initial prompt

For realistic human voice interactions, we adopted the OpenAI Whisper model for speech-to-text and text-to-speech (TTS) tasks. We selected GPT-3.5-turbo as our primary LLM after comparative testing with GPT-4 and Mistral-7B-OpenOrca showed similar performance in command interpretation but faster response times with GPT-3.5-turbo. Our LLM prompt employs a structured three-component design: role definition, skill specification, and JSON response formatting. The prompt explicitly defines available skills (e.g., Follow(), Fetch(), SymmetricalFollow()) and requires standardized JSON responses such as “Skill”: “Follow()”, “Description”: “I’ll follow your arm movement to help push the object together.” This ensures consistent command interpretation and seamless integration with our control pipeline. The complete prompt structure is detailed in the appendix (Figure 9).

To optimize the robot assistant’s understanding of its role and objectives, we implemented a set of predefined rules and instructions as an initial prompt for the LLM. The initial prompt configures the LLM as an AI assistant designed to aid a robot arm in task execution. It instructs the LLM to generate scripts based on the user’s spoken commands, adhering to a specific JSON format: Script: “Skill: Write the function here.“, “Description: Include a necessary description about this skill, as if you are talking to the user directly. Use ‘you’ to address the user.” The robot assistant is equipped with a comprehensive list of available skills from the skill database to enable matching of user commands with appropriate functions. The LLM is programmed to provide user feedback on its actions through the “Description” field in the JSON script. When a user’s command corresponds to a known skill, the LLM generates the relevant script. In cases where no match is found, the assistant generates a script with an empty function and a description indicating that no action will be taken. This structured approach to the initial prompt ensures the LLM-aided robot assistant’s ability to interpret user commands and provide meaningful feedback, which facilitates a more seamless and effective interaction between the human operator and the embodied AI system in bimanual handling tasks. Additionally, this safety check effectively addresses potential conflicts or misinterpretations between the LLM’s voice command interpretation and the predefined skill base. The LLM is configured with a structured prompt (see Figure 9) that defines available skills and expected response formats. This ensures consistent interpretation of operator commands and appropriate skill selection. The system provides immediate feedback through natural language responses, confirming command understanding before execution.

#### 4.1.3 Skills

There are two types of skills: autonomous and real-time skills. Autonomous skills are executing actions in series and exiting when the whole action is done, such as Handover()—handover an object to the master arm; Approach()—move the arm to approach an object (e.g., for listing objects together); Fetch()—grab an object and bring it to the master arm. Real-time skills are continuous motions and exiting when the user gives the stop command, like Follow()—follow the master robot arm (e.g., for pushing together); SymmetricalFollow()—act a mirror behavior of the master robot arm; Move(distance, direction)—move the arm (ask user for distance in meters and direction: “+x”, “-x”, “+y”, “-y”, “+z”, “-z”). Each skill includes parameter validation and safety checks to ensure reliable operation.

### 4.2 Training protocol

**FIGURE 3:**
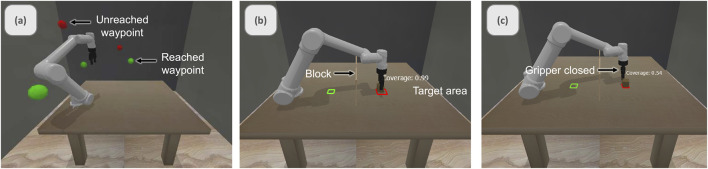
Single arm training tasks: **(a)** target reaching, **(b)** pick-and-place, and **(c)** pushing.

We developed a structured training protocol to ensure consistent operator proficiency across all experimental conditions. Each participant completed three increasingly complex tasks: target reaching, pick-and-place, and pushing operations (Figure 3).

This progressive training approach helped participants develop fundamental skills before attempting more complex bimanual operations. In the target-reaching task, the goal was to navigate to the red waypoints. The pick-and-place task required participants to use the gripper to grasp a square block and transport it to a target area while avoiding a vertical barrier. The pushing task involved pushing an object into a designated target area. Participants were required to complete the tasks within 4 and 3 min, respectively.

### 4.3 Experiments procedure

**FIGURE 4:**
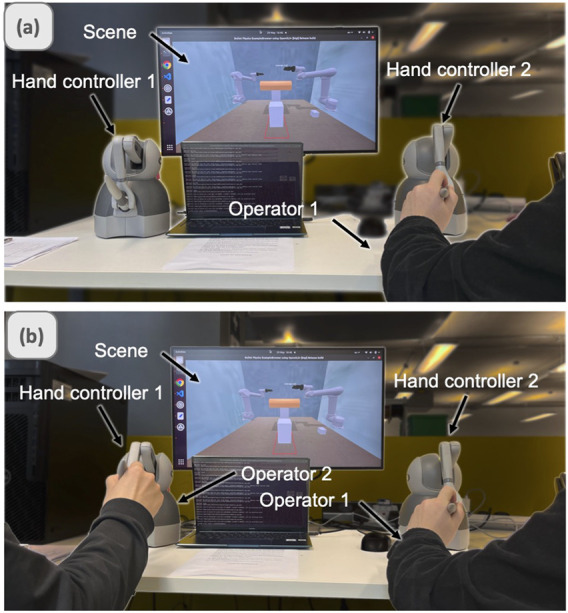
Experimental setup for bimanual teleoperation: **(a)** SPB and **(b)** Dyadic configurations.

The experimental task required coordinated bimanual manipulation to transport an object to a designated platform (Figure 4). We evaluated three teleoperation patterns: SPB, Dyadic, and BTLA, with participants experiencing each mode in counterbalanced order. In the baseline SPB condition, participants controlled both robotic arms simultaneously using haptic devices, representing traditional teleoperation approaches. The dyadic teleoperation condition paired participants with a trained operator, simulating collaborative control scenarios. BTLA condition enabled participants to control the master arm directly while commanding the assistant arm through voice instructions. After each trial, participants completed the NASA-TLX questionnaire and provided feedback on their experience. Three types of teleoperation were tested in randomized order to tackle learning effects.

**FIGURE 5:**
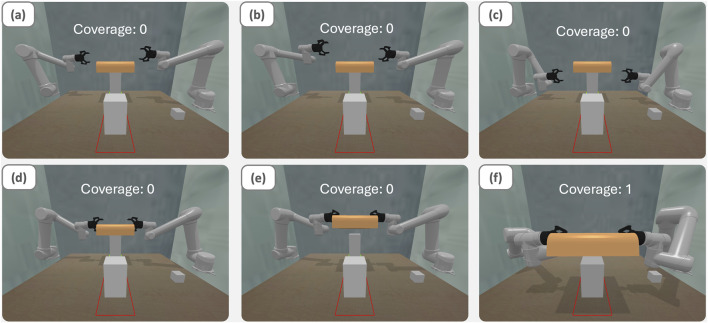
Illustration of the execution of BTLA on object transferring tasks: **(a)** initial state **(b)** move the left arm only without following command **(c)** right arm is controlled by BTLA with symmetrical following behavior **(d)** go to pick up position simultaneously **(e)** grab the object **(f)** collaborate with BTLA moving the object to the specific place.

The experimental task involved coordinated manipulation of a large object, requiring precise control during grasping, transport, and placement phases. As illustrated in Figure 5, successful completion demanded stable bimanual coordination to move the object to a designated target location while maintaining proper orientation and avoiding collisions. Figures 5a–d shows the motion from the start position to the grasp position. Figures 5e,f shows the motion to the appointed platform.

### 4.4 Assessment

We defined a successful trial using three criteria: successful simultaneous object grasping by both arms, stable object transport without drops or collisions, and accurate placement with at least 70% coverage of the target area. For each teleoperation pattern, we recorded multiple trials to assess the consistency and reliability of performance.

System usability and operator experience were assessed through two complementary questionnaires. The first evaluated the quality of human-robot interaction across multiple dimensions, including interface naturalness, operator satisfaction, perceived system intelligence, and overall usability. The second utilized the NASA-TLX to measure operator workload across six dimensions: mental demand, physical demand, temporal demand, performance, effort, and frustration (Figure 8). This standardized assessment tool has been widely validated in human-machine interaction studies (Hart and Staveland, 1988) and provides robust metrics for comparing different teleoperation approaches.

## 5 Results and discussions

### 5.1 Performance metrics

**FIGURE 6:**
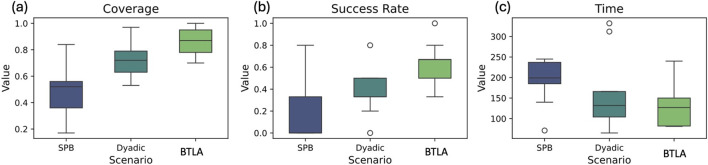
Box plots for performance **(a)** coverage (p<0.05) , **(b)** success rate (p<0.05) , **(c)** time (p=0.117) among all subjects for three experimental scenarios SPB, Dyadic, and BTLA.

To evaluate the effectiveness of the BTLA, we compared its performance with the Dyadic and SPB scenarios using three metrics: coverage, success rate, and task completion time, as shown in Figure 6. The BTLA scenario demonstrated the highest mean coverage (0.861) and success rate (0.627) among the three scenarios, suggesting that the BTLA system is more effective in completing tasks and covering a larger portion of the task space compared to the Dyadic and SPB scenarios. The Kruskal–Wallis test was performed to assess the statistical significance of the differences in coverage (p=0.003) and success rate (p=0.004) among the patterns, and the results indicate that the differences in these metrics among the scenarios are statistically significant. Although the BTLA scenario exhibited faster task completion times compared to the other patterns, the differences were not statistically significant based on the Kruskal–Wallis test, which yielded a p-value of 0.117 for the time metric. To identify specific group differences, we conducted post-hoc pairwise comparisons using the Dunn test with Bonferroni correction. For coverage, BTLA significantly outperformed both SPB (p<0.001) and Dyadic (p=0.032) conditions, while the difference between Dyadic and SPB was not significant (p=0.089) . Similarly, for success rate, BTLA showed significant improvements over SPB (p<0.001) and Dyadic (p=0.045) , with no significant difference between Dyadic and SPB (p=0.156) . These results confirm that BTLA provides the most substantial performance gains compared to traditional teleoperation approaches.

**FIGURE 7:**
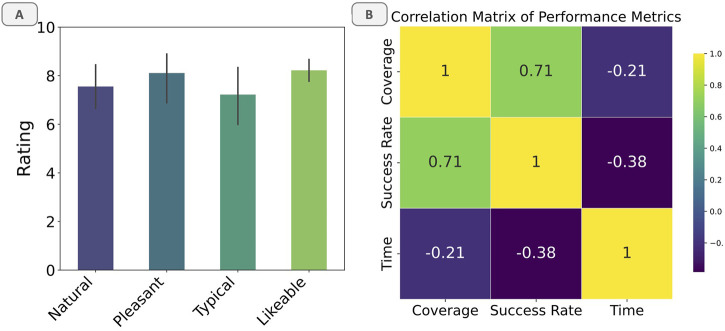
**(a)** Likert Scale Ratings. **(b)** Correlation matrix of performance metrics.

Furthermore, a correlation analysis was conducted to examine the relationship between coverage and success rate (see Figure 7). The analysis revealed a strong positive correlation (0.71) between the two metrics, indicating that higher coverage is associated with higher success rates. This finding suggests that the BTLA system’s ability to cover a larger portion of the task space contributes to its higher success rates in completing tasks compared to the Dyadic and SPB patterns.

### 5.2 Subjective assessment

**FIGURE 8:**
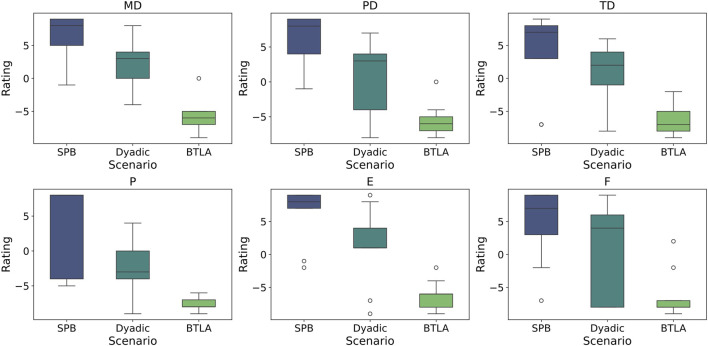
Boxplots for NASA-TLX results among all subjects for three experimental patterns: SPB, Dyadic, and BTLA, respectively. Rated aspects from NASA-TLX: mental demand (MD), physical demand (PD), temporal demand (TD), performance (P), effort (E), and frustration (F). (all metrics: p<0.05 ).

For all NASA-TLX metrics (mental demand (MD), physical demand (PD), temporal demand (TD), performance (P), effort (E), and frustration (F)), the BTLA pattern exhibited the most favorable ratings, with lower demands, effort, and frustration, as well as better perceived performance compared to the Dyadic and SPB patterns as shown in Figure 8. In contrast, the SPB pattern appeared to be the most challenging, with higher demands, effort, and frustration, and lower perceived performance. The Dyadic pattern fell between the BTLA and SPB, indicating moderate levels of demands, effort, frustration, and performance.

The Kruskal–Wallis test results revealed statistically significant differences among the three patterns for all metrics, with the test statistics being 17.974 for MD (p≪0.001) , 14.701 for PD (p=0.001) , 12.276 for TD (p=0.0002) , 15.723 for P (p≪0.001) , 14.228 for E (p=0.0001) , and 11.018 for F (p≪0.001) . The p-values for all metrics were less than 0.001, providing strong evidence against the null hypothesis of no difference among the patterns. Over 40% of participants reported that their performance was limited by the restricted 2D camera view. This limitation was due to either a loss of depth perception, making it difficult to discern spatial relationships, or because the images were partially obstructed.

In summary, experiment results showed marked improvements in task performance and lowered operator workload versus conventional methods, with natural language interpretation and adaptive assistance proving critical for complex manipulations. However, the integrated voice processing pipeline—comprising speech-to-text conversion via Whisper, intent interpretation through GPT-3.5-turbo, and skill dispatch—introduces a measurable latency, which may impede real-time responsiveness during high-speed bimanual coordination tasks such as dynamic obstacle avoidance. Furthermore, validation remains confined to simulated environments using PyBullet; deployment on physical hardware necessitates addressing critical challenges, including sensor noise robustness and unmodeled dynamics (e.g., joint friction and cable effects). Future work includes three key directions: (1) broadening autonomous behaviors and refining real-time autonomy adaptation to boost flexibility; (2) exploring mutual adaptation between operators and the system during extended use to optimize collaboration; and (3) extending BTLA’s application to diverse robotic platforms and real-world scenarios to strengthen practical relevance.

## 6 Initial prompts

See Figure 9.

**FIGURE 9:**
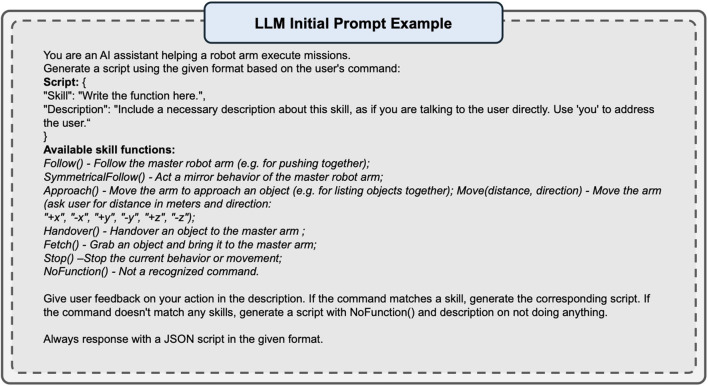
An example of LLM initial prompt: textual description of the mission and skills.

## Data Availability

The original contributions presented in the study are included in the article/supplementary material, further inquiries can be directed to the corresponding author.
